# Comparative Analysis of Correlative Modeling Methods in Predicting North American Bird Abundance

**DOI:** 10.1002/ece3.71982

**Published:** 2026-08-03

**Authors:** Jazmín Escobar‐Luján, Fabricio Villalobos, Adolfo G. Navarro‐Sigüenza, Carlos Yañez‐Arenas

**Affiliations:** ^1^ Posgrado en Ciencias Biológicas Universidad Nacional Autónoma de México, Ciudad de México Ciudad de México Mexico; ^2^ Laboratorio de Ecología Geográfica Universidad Nacional Autónoma de México Chuburná Yucatán Mexico; ^3^ Red de Biología Evolutiva Instituto de Ecología Xalapa Veracruz Mexico; ^4^ Museo de Zoología – Departamento de Biología Evolutiva, Facultad de Ciencias Nacional Autónoma de México, Ciudad de México Ciudad de México México

**Keywords:** ecological niche models, environmental suitability, North American birds, prediction of abundance

## Abstract

Over the past decade, correlative ecological niche models (ENMs) have been increasingly used to infer species abundance through the estimation of continuous scores, which can be interpreted as environmental suitability. However, little attention has been drawn to the assumptions and conceptual differences between these methods and how they affect abundance predictions. Here, we address this gap by comparing the performance of nine ENM methods, including some recently proposed approaches, in predicting the abundance of North American birds using standardized count data. Relationships between abundance and environmental suitability were generally positive, with average correlation coefficients greater than 0.18. This implies that environmental suitability can partially explain abundance, as it is likely influenced by other factors such as biotic interactions and dispersal constraints. Although no method proved to be significantly superior, newer modeling approaches (e.g., MaxLike and Gaussian models) showed higher average correlations compared to older ones (e.g., Boosted Regression Trees and Random Forest), underscoring the comparable performance across modeling approaches. Results were consistent across taxonomic groups (e.g., Passerellidae), an indication that the observed patterns are robust within specific clades. Furthermore, no significant differences were found between models using complete distribution data versus breeding area data, or annual versus seasonal environmental variables, highlighting that simpler data inputs may be enough for abundance prediction in many contexts. Taken together, these findings reinforce the potential of ENMs to provide reliable insights into species abundance patterns.

## Graphical abstract

This study compares nine ecological niche model (ENM) methods to predict North American bird abundance and finds positive correlations between environmental suitability and abundance. Results highlight the potential of ENMs for abundance inference, with newer methods showing good performance and demonstrating that simpler data inputs can be effective for many applications.

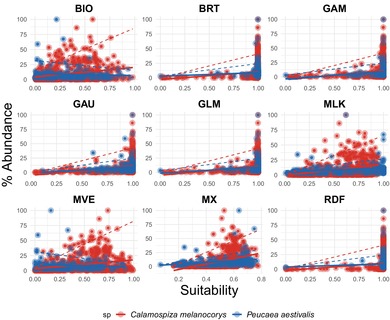

## 1 Introduction

Species abundance is a primary indicator of habitat quality, reflecting essential ecological factors such as reproductive success, longevity, carrying capacity, and extinction risk. A high abundance of a species is often associated with favorable conditions for its persistence (Hobbs and Hanley 1990; Mace et al. 2008). It thus represents a valuable parameter for conservation efforts, including reintroductions, population connectivity assessments, and the establishment of natural protected areas (Fischer and Lindenmayer 2007). However, collecting comprehensive abundance data poses significant challenges, as it is time‐consuming, logistically complex, and financially demanding. In contrast, presence‐only data are more readily available at large spatial scales, facilitated by biodiversity repositories, citizen science initiatives, and remote sensing technologies (Johnston et al. 2015; Newbold 2010). This accessibility has increased reliance on correlative presence‐based methods, such as ecological niche models (ENMs), for inferring species abundance (Peterson et al. 2011).

ENMs generate continuous values interpreted as environmental suitability, with higher values indicating more favorable conditions for species persistence and proliferation (Gil and Lobo 2017). The strength of this relationship has been observed to vary considerably, with correlations ranging from strong positive to weak or null (Dallas et al. 2017; Osorio‐Olvera et al. 2020; Pironon et al. 2015; Santini et al. 2019; Weber et al. 2017; Yañez‐Arenas et al. 2014). Notably, many of these studies have identified a triangular relationship whereby low suitability values are consistently associated with low abundance. In contrast, high suitability values correspond to a broad range of abundance levels. This pattern may be attributed to the fact that ENMs predominantly rely on abiotic variables and frequently exclude factors such as biotic interactions, dispersal limitations, and anthropogenic impacts, which can mediate the abundance‐suitability relationship (Osorio‐Olvera et al. 2019; Pironon et al. 2018). Furthermore, spatial biases in presence records and variability in the quality of abundance data introduce additional complexity to these inferences (Knouft 2018; Yañez‐Arenas et al. 2014). Lastly, discrepancies in the mathematical assumptions that underpin disparate modeling techniques may impact their capacity to predict abundance, a factor that has not been subjected to a comprehensive evaluation (Sillero et al. 2023).

In accordance with their underlying mathematical assumptions, correlative methods can be classified into three principal categories: statistical models, similarity/envelope models, and machine learning methods. Each of these categories is characterized by a distinct conceptual foundation, which in turn affects their performance in predicting abundance (Weber et al. 2017). Statistical models, such as generalized linear models (GLMs) and generalized additive models (GAMs), are designed to identify straightforward relationships between species presence and environmental predictors (Franklin 2012). These models are consistent with ecological theory, which posits that species performance is optimal within a specific environmental range and declines outside of it. This renders them especially useful for studies aiming to elucidate the manner in which abundance responds to environmental gradients (Angilletta 2009). In contrast, similarity or envelope models, such as BIOCLIM and Minimum Volume Ellipsoid (MVE), are designed to delineate the environmental space occupied by a species, assuming that suitability decreases with distance from optimal conditions (Maguire 1973; Martínez‐Meyer et al. 2013). Although these models may not explicitly capture species tolerance curves, they are advantageous when the objective is to identify areas with broad suitability, even if their relationship with abundance at specific locations may be weaker (Tôrres et al. 2012). Machine learning methods, such as Random Forest and MaxEnt, offer greater flexibility in modeling complex and nonlinear relationships between species presence and environmental variables (Franklin 2012; Phillips et al. 2006; Weber et al. 2017). However, their predictive performance depends heavily on the quality of the input data. Spatial biases and limited sample sizes, for instance, can lead to overfitting and unreliable estimates (VanDerWal et al. 2009; Yañez‐Arenas et al. 2014). Recent advances have improved the interpretability of machine learning models by providing tools such as variable importance metrics, partial dependence plots, and spatial decomposition (e.g., Olden et al. 2008; Elith et al. 2008). Nevertheless, the complex nature of these algorithms still limits their transparency relative to simpler statistical models, which can make ecological interpretation more difficult.

Despite the growing diversity of ENM approaches, most empirical research on the abundance‐suitability relationship has focused on a limited number of methods (Phillips et al. 2006; Weber et al. 2017). This has resulted in a lack of robust assessment of their effectiveness, particularly given the emergence of new algorithms with distinct assumptions and capabilities. Among these newer approaches, Gaussian models and MaxLike have demonstrated potential in addressing some of the limitations of traditional methods (Fitzpatrick et al. 2013; Golding and Purse 2016). Gaussian models, which are classified as statistical methods, employ flexible processes to capture complex, continuous relationships between environmental predictors and species presence. This allows them to offer ecologically plausible responses while accounting for uncertainty in predictions (Fitzpatrick et al. 2013). MaxLike is a hybrid approach that combines statistical principles with machine learning optimization. It estimates occurrence probabilities from presence‐only data by maximizing the likelihood, making it particularly effective for datasets subject to inherent biases or incomplete detection information (Royle et al. 2012). The capacity of these methods to provide insight into species abundance remains understudied, underscoring the necessity for a comprehensive evaluation of their performance in conjunction with more established techniques.

To evaluate the performance of ENM methods in predicting abundance, a study system with comprehensive species distribution data is necessary. It should include presence records, often available in repositories, as these are typically the most accessible data for users, alongside reliable and high‐quality abundance data. Although these approaches are frequently referred to as Species Distribution Models (SDMs) or Habitat Suitability Models (HSMs), we use the term Ecological Niche Models (ENMs) in this study to highlight our conceptual focus on linking species' environmental requirements to abundance patterns, following the niche‐based framework proposed by Peterson and Soberón (2012). In this study, we evaluate the performance of nine correlative ENM methods, including recently proposed approaches, in predicting species abundance. Using North American birds as a model system, we assess how theoretical assumptions and methodological choices influence the relationship between modeled environmental suitability and observed abundance. To exemplify the records used and illustrate part of the results, we included *Calamospiza melanocorys* (Lark Bunting) and *Peucaea aestivalis* (Bachman's Sparrow). However, the study focuses on the general patterns observed across all species analyzed. Our results offer a large‐scale assessment of the predictive reliability of different modeling methods, providing critical insights into their application for ecological research and conservation planning.

## 2 Materials and Methods

### 2.1 Species Data

Our study system consisted of North American birds, and the selected species were those in which more than 80% of their distribution range overlapped with the sampling area of the North American Breeding Bird Survey (BBS, Table S1). We chose this study system because the abundance information is available to address the abundance‐suitability relationship. The BBS is a standardized sampling program that has been in operation for over 60 years (https://www.usgs.gov/centers/eesc/science/north‐american‐breeding‐bird‐survey; accessed on August 2021). This sampling protocol entails the implementation of annual transects along fixed 39.4 km routes, wherein expert bird observers conduct censuses of all avian species and individuals at designated counting points situated at one‐kilometer intervals, with observations lasting 3 min per point. The BBS has compiled decades of data, enabling the study of long‐term trends. Additionally, it covers extensive regions of North America, offering invaluable information on a wide range of species and habitats (Peterjohn and Sauer 1999). We selected this species set because there is abundant information available for most of their distribution range. We obtained the georeferenced occurrences for each bird species from the Global Biodiversity Information Facility (https://www.gbif.org; accessed on August 2021) database. GBIF is a global infrastructure that provides open access to a vast amount of biodiversity data, thereby enabling large‐scale studies. The data set comprises species occurrence records derived from a wide range of sources, including scientific institutions and citizen science projects. However, the quality and format of the data can vary significantly across sources, which could complicate the process of analysis and interpretation (Robertson et al. 2014). We refined the downloaded occurrences for each species based on their reported distribution ranges in the Cornell Lab of Ornithology (https://birdsoftheworld.org; accessed on August 2021) and BirdLife (https://www.birdlife.org/; accessed on August 2021) databases. For migratory species, we created a second additional presence dataset consisting of records from their known and reported breeding areas in the databases mentioned earlier (Table S1). We generated this new presence dataset to assess whether the information from the breeding area can reflect the relationship between environmental suitability and abundance since it is where the reproduction and nesting of migratory birds occur. To reduce the risk of spatial bias and overfitting in the models (Varela et al. 2014), we filtered presence records at 40 km using the “CoordinateCleaner” package (Zizka et al. 2019). This approach was necessary because the sampling of the records used tends to be concentrated in areas accessible by roads. This distance was selected based on the length of the BBS monitoring transect.

**FIGURE 1:**
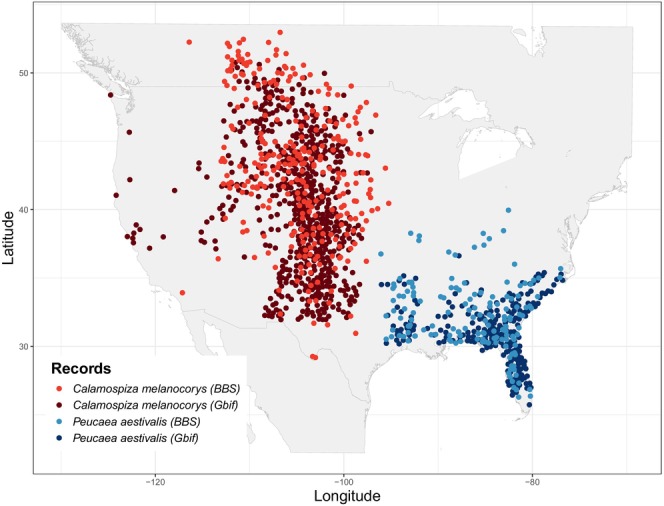
Spatial distribution of presence records and abundance of Lark Bunting ( *Calamospiza melanocorys* ) and Bachman's Sparrow ( *Peucaea aestivalis* ) used in the study. The dark red and dark blue dots represent the GBIF (Global Biodiversity Information Facility) presence records for Lark Bunting and Bachman's Sparrow, respectively, which were used to construct the model. The light red and light blue dots correspond to the locations for each species where abundance data were collected from the North American Breeding Bird Survey (BBS).

In addition, we obtained abundance data from the BBS by obtaining tables of abundance counts at the route level along with their metadata for the period from 1966 to 2021. We estimated the average number of individuals across the years when the route was surveyed, and this value was used as a measure of abundance. We considered the species that were detected at least once on the route. Figure 1 illustrates the presence records and locations with abundance information for the species *Calamospiza melanocorys* (Lark Bunting) and *Peucaea aestivalis* (Bachman's Sparrow), offering an example of the spatial distribution of the data used in the analysis.

### 2.2 Environmental Data

We downloaded climatic variables from Worldclim version 2.1 (Fick and Hijmans 2017) at a spatial resolution of 10 min. Worldclim is a global database that provides high‐resolution interpolated climate layers, offering historical data on temperature, precipitation, and other climatic factors. The data set encompasses annual trends and deviations from 1970 to 2000, offering a comprehensive representation of climatic variations. We decided to evaluate whether the temporality of environmental variables (i.e., whether they were annual or aligned with the breeding season when abundance data were collected) might influence the abundance‐suitability relationship. To achieve this, we created two sets of climatic variables: the first set comprised the 19 bioclimatic variables (derived from annual temperature and precipitation values), while the second set included climatic variables corresponding to the breeding season (May–August; Martínez‐Meyer et al. 2004; Nakazawa et al. 2004). These months do not always coincide with the breeding periods of resident species, which are often poorly understood (Gómez et al. 2016). This second set of variables was generated from the monthly values of maximum, minimum, and average temperatures (tmax, tmin, tavg, respectively) and precipitation (prec) using the “terra” package (Hijmans et al. 2024).

Both sets of climatic variables (annual and breeding season values) were clipped using a polygon representing a hypothesis of historical accessibility for each species, corresponding to the M area in the BAM diagram (Soberon and Peterson 2005). Calibrating the models within the M area prevents predictions in regions historically inaccessible to the species and reduces the risk of inflating evaluation metrics (Barve et al. 2011; Cooper and Soberón 2018). For each species, the M area was delineated by intersecting WWF terrestrial ecoregions (Olson et al. 2001) with the presence records using the “terra” package in R (Hijmans et al. 2024). Similarly, for migratory species, a specific M area for the breeding area was defined based on the ecoregions containing occurrence records from that area. Both sets of climatic variables were then clipped using these respective delimitations. As a result, four sets of climatic variables were obtained for migratory species: two corresponding to the species' range‐wide M area and two corresponding to the breeding M area. For non‐migratory species, only two sets of climatic variables were generated, corresponding to the range‐wide M area.

For each set of climatic variables per species, we performed a principal component analysis (PCA) using the ‘ENMGadgets’ package (Barve and Barve 2013) to reduce multicollinearity and dimensions in environmental variables. We chose PCA because it allows for the transformation of correlated environmental variables into a smaller set of uncorrelated components, simplifying the modeling process by reducing redundancy among predictor variables. We retained the first three components that explained approximately 90% of the total variance as predictor variables for modeling the ecological niche of each species. Using only these components as predictors reduces the risk of overfitting while maintaining a meaningful representation of the environmental variation relevant to the species' ecological niches.

### 2.3 Estimating Environmental Suitability

We used nine ENM methods that employ presence records and environmental information to estimate the environmental suitability of each species. We included statistical, similarity/envelope, and machine learning methods. The statistical methods included generalized linear models (GLM; McCullagh and Nelder 1989), generalized additive models (GAM; Hastie and Tibshirani 1990), and Gaussian models (GAU; Golding and Purse 2016). The similarity or envelope methods used were BIOCLIM (Busby 1991) and minimum volume ellipsoids (MVE; Van Aelst and Rousseeuw 2009). The machine learning methods were MaxEnt (MX; Phillips et al. 2006), MaxLike (MLK; Royle et al. 2012), Random Forest (RDF; Breiman 2001), and Boosted Regression Tree (BRT; De'ath 2007).

Presence records were divided into four spatially independent blocks using the ENMEval package (Muscarella et al. 2014), ensuring spatial independence between calibration and evaluation datasets. In each iteration, two diagonally opposite blocks were used for calibration, and the other two for evaluation. The process was repeated by switching the calibration and evaluation blocks, ensuring that all records contributed to both calibration and evaluation in a spatially independent manner. We evaluated each model based on the AUC ratio of the partial ROC technique and the omission rate, allowing a 5% error in both metrics (Peterson et al. 2008). All models were calibrated and evaluated using the same input data (presence records and background environmental points) and the same parameters as much as possible (see Appendix S1). This ensured that any differences in results could be attributed solely to the intrinsic characteristics of the methods themselves.

### 2.4 Abundance‐Environmental Suitability Relationship

We evaluated the relationship between abundance and environmental suitability derived from ENM methods using Spearman correlations, calculated with the “cor.test” function of the stats package. Additionally, we conducted Kruskal–Wallis and Dunn tests (after verifying the normality assumption) to determine if there were differences in correlation values (rho's) between three modeling decisions: the spatial representation of occurrences (whether they correspond to the entire range or only of the reproductive region, in the case of migratory species), the temporal representation of environmental variables (whether they are annual variables or coincide with the breeding season—May to August—in which the abundance data were obtained), and the modeling method. All statistical analyses were performed using the R language (R Core Team 2022).

## 3 Results

**FIGURE 2:**
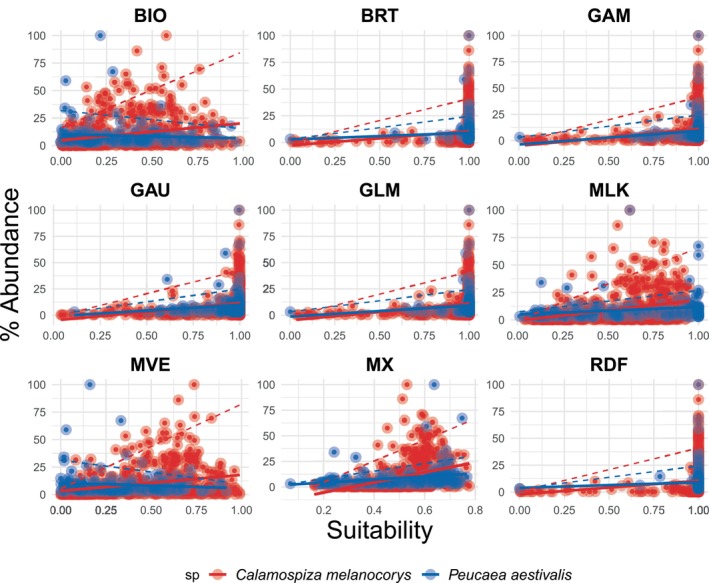
Relationship between abundance and environmental suitability estimated using nine different methods for the Lark Bunting ( *Calamospiza melanocorys* ) and Bachman's Sparrow ( *Peucaea aestivalis* ). The red dots correspond to the Lark Bunting and the blue dots to the Bachman's Sparrow. The regression lines represent the relationships fitted using ordinary least squares regression (solid line) and the 97.5th percentiles of the quantile regressions (dashed line). BIO, BIOCLIM; BRT, Boosted Regression Tree; GAM, generalized additive models; GAU, Gaussian models; GLM, generalized linear models; MLK, MaxLike; MVE, minimum volume ellipsoids; MX, MaxEnt; RDF, Random Forest.

**FIGURE 3:**
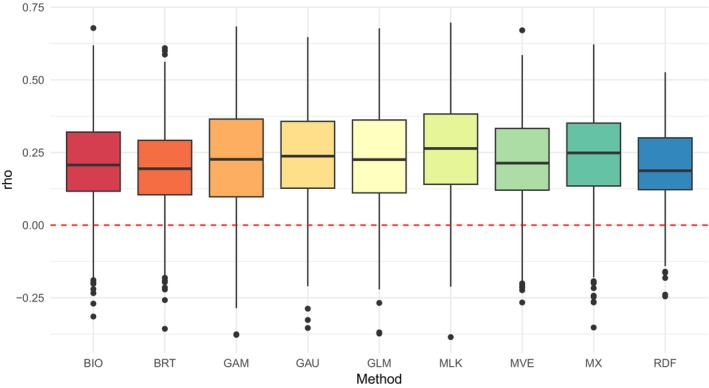
Rho values of the relationships between the suitability modeled by the methods and abundance. BIO, BIOCLIM; BRT, Boosted Regression Tree; GAM, generalized additive models; GAU, Gaussian models; GLM, generalized linear models; MLK, MaxLike; MVE, minimum volume ellipsoids; MX, MaxEnt; RDF, Random Forest.

The evaluation of ecological niche models showed that 86.4% achieved statistically significant performance, with an average AUC ratio of 1.05. Generally, the relationships between environmental suitability and abundance were triangular and non‐linear (Figure 2). We found at least one positive correlation between the modeled suitability obtained by one of the methods and the observed abundance in 90 of the 118 species analyzed, with 74.4% of these correlations being statistically significant. All evaluated methods demonstrated significant relationships between abundance and environmental suitability for more than 60% of the species analyzed. MaxLike and Gaussian models exhibited the highest rates of significant models (72% each) and achieved the strongest correlations, with mean rho values of 0.255 and 0.236, respectively. However, these methods also presented the highest standard deviations in rho (0.193 and 0.194). In contrast, MVE showed a moderate mean rho value (0.217) but demonstrated lower variability, with a standard deviation of 0.181 (Table 1 and Figure 3). The general pattern of method performance remained consistent when examined within species of a family (Passerellidae), with the same methods showing the highest correlations between abundance and suitability (Figure S1).

**TABLE 1:** Methods with significant relationships between suitability and abundance.

Method	Percentage of significant relationships	Percentage of positive relationships	Mean *ρ*	SD *ρ*
MLK	72.0	81.8	0.255	0.193
GAU	72.0	83.5	0.236	0.194
MX	71.5	76.2	0.223	0.198
GAM	67.2	77.5	0.218	0.196
MVE	66.2	81.2	0.217	0.181
GLM	69.0	78.0	0.215	0.196
RDF	63.7	80.8	0.203	0.148
BIO	66.5	76.0	0.202	0.173
BRT	64.5	75.5	0.189	0.174

*Note:* Percentage of models with significant and positive relationships. Mean *ρ* values and their deviation.

Abbreviations: BIO, BIOCLIM; BRT, Boosted Regression Tree; GAM, generalized additive models; GAU, Gaussian models; GLM, generalized linear models; MLK, MaxLike; MVE, minimum volume ellipsoids; MX, MaxEnt; RDF, Random Forest.

We found significant differences in the abundance‐suitability correlations among the different methods (*ꭓ* ^2^ = 31.4, *p* = 1.20 × 10^−4^). Specifically, BRT showed significantly lower rho values compared to MLK (*z* = −4.48, *p* = 1.00 × 10^−3^) and GAU (*z* = −3.32, *p* = 0.03). Additionally, MLK had higher rho values than RDF, with a significant difference observed (*z* = 3.807, *p* = 4.00 × 10^−3^). These findings highlight that most of the significant differences in abundance‐suitability correlations were observed between machine learning methods, such as MLK, BRT, and RDF. For the 94 migratory bird species analyzed (representing 79.6% of all species), we found no significant differences in the correlations between abundance and environmental suitability in models generated using annual or reproductive season variables (*ꭓ* ^2^ = 2.77, *p* = 0.10). Similarly, no significant differences were observed when correlations between abundance and environmental suitability were based on presence records representing either the full distribution range or only the reproductive areas (*ꭓ* ^2^ = 100.3, *p* = 0.31).

## 4 Discussion

In this study, we aimed to evaluate the performance of correlative ENM methods using only occurrence data in predicting North American bird abundance. We found positive relationships between abundance and environmental suitability across the methods studied, indicating consistent patterns despite methodological differences. This suggests that various ENM methods can partially capture spatial variations in species abundance. The findings are consistent with previous studies (de la Fuente et al. 2021; Lunghi et al. 2018; Osorio‐Olvera et al. 2020; Weber et al. 2017; Williams et al. 2021). Therefore, our evaluation revealed significant abundance‐suitability relationships by employing methods previously evaluated individually, whose performance is confirmed when applied to a large set of species. In addition to the methods addressed in these previous studies, we incorporated novel methods not considered before in abundance prediction evaluations (MaxLike and Gaussian models), which interestingly had high performance (see below). Our findings differ from previous research as we observed correlations between suitability and abundance, which were absent in those studies (Dallas et al. 2017; Santini et al. 2019). However, these studies encountered challenges due to uncertainties and biases in their data and methodologies. For instance, inadequate control over the spatial resolution of climate variables in relation to abundance data resolution may have impacted the relationships between abundance and environmental suitability (Knouft 2018; Soberón et al. 2018). Ensuring the reliability of abundance data is crucial to avoid including data altered by human activities that do not reflect ecological processes, such as introducing exotic individuals in fishing activities (Knouft 2018). This study addressed these concerns by carefully considering spatial resolution and utilizing high‐quality abundance information, as recommended in previous studies (Osorio‐Olvera et al. 2020). Additionally, we used presence records from repositories, the most commonly used source of information in correlative niche modeling. It is important to acknowledge these data sources' uncertainties and biases and adopt best practices outlined in previous studies (Araújo et al. 2019). A key limitation of these data is imperfect detection, which can bias suitability estimates through the uneven recording or omission of occurrences (Guillera‐Arroita et al. 2015). Although our broad‐scale approach did not allow us to address this directly, we acknowledge it as a source of uncertainty, and future studies should consider detection‐adjusted models when data permit, especially at finer scales.

The positive abundance‐suitability relationship stems from the species' ecological niche concept, where abundance responds to local environmental conditions. Typically, species inhabit areas with suitable abiotic conditions, conducive to growth, and where biotic interactions allow population persistence and colonization (Soberón 2007; Soberon and Nakamura 2009). Favorable environments enhance the physical condition of individuals, promoting their survival and reproductive success, thus reducing the risk of extinction and potentially increasing abundance (Acevedo et al. 2017; Araújo et al. 2002; Brambilla and Ficetola 2012; de Moraes Weber and Grelle 2012; Fitzgerald‐Dehoog et al. 2012; Morrison et al. 2012; VanDerWal et al. 2009). Moreover, environmental suitability correlates with population carrying capacity, often higher in areas with high suitability values (Thuiller et al. 2014). Our models estimated climatic suitability using long‐term climatic averages and presence records aggregated over decades. As such, the suitability estimates reflect climatic suitability rather than broader environmental suitability. This distinction is important because while climatic suitability captures broad‐scale, climate‐driven constraints, it does not account for fine‐scale variation in abundance that may arise from land use, habitat quality, or temporal fluctuations. Consequently, although we observed positive abundance–suitability relationships, these relationships exhibited a triangular pattern (Acevedo et al. 2017), indicating that high climatic suitability does not necessarily translate to high abundance. This pattern may result from the inability of ENMs to include factors such as biotic interactions, dispersal limitations, anthropogenic impacts, or local characteristics, as discussed by other authors (Carrascal et al. 2015; Tôrres et al. 2012; VanDerWal et al. 2009; Weber et al. 2017). This suggests that species abundance can vary widely in areas of high environmental suitability. For instance, the abundance of Lark Buntings is influenced by local vegetation structure, such as shrub cover and ground conditions, while Bachman's Sparrows respond to habitat characteristics and fire regimes, including burn timing and extent (Greer et al. 2016; Jones et al. 2014; Tucker et al. 2004). These cases underscore that areas deemed suitable by ecological niche models (ENMs) may lack key local factors required for high abundance. Additionally, abundance‐suitability relationships may be shaped by spatial bias in occurrence records and abundance data quality (Knouft 2018; Soberón et al. 2018; Yañez‐Arenas et al. 2014). We addressed this issue by applying spatial filtering to presence data (Varela et al. 2014) and by using standardized BBS datasets. However, intrinsic ecological processes, such as biotic interactions and metapopulational dynamics, may influence abundance in ways not captured by the models (Osorio‐Olvera et al. 2020, 2016; Weber et al. 2017). Thus, while our macroecological approach identifies broad environmental constraints on abundance, it may not fully capture the complexity of local abundance variation, highlighting a key limitation when extrapolating local patterns from coarse‐scale suitability models.

The discrepancies in abundance‐suitability relationships among ENM methods stem from their methodological foundations. Ideally, correlative niche modeling should reflect the intrinsic relationship between species presence and suitability based on environmental variables. However, each method employs distinct approaches to establish this relationship (Soberon and Nakamura 2009). While similarity or envelope methods like BIO or MVE assume unimodal relationships based on ecological theory (Qiao et al. 2016), statistical methods like GAM or GAU adjust similar responses from the data (Franklin 2012). Conversely, machine learning methods like MX, BRT, and RDF capture complex non‐linear relationships, characterizing species' spatial distributions (Elith et al. 2011; Peterson and Soberón 2012). Thus, the relationships between abundance and suitability estimated by these methods can be affected differently. For instance, envelope methods rely on the proper characterization of species' fundamental niche (Osorio‐Olvera et al. 2019), while machine learning models are sensitive to spatial bias in presences (Yañez‐Arenas et al. 2014), necessitating strategies like spatial filtering and bias covariate inclusion (Boria et al. 2014; Phillips et al. 2009). Understanding these methodological nuances is crucial when using suitability estimation outputs as proxies for species abundance. Past evaluations, such as those for jaguar density (Tôrres et al. 2012), often did not account for overfitting in machine learning models and found BIO to perform best. In our study, we found no significant differences between envelope and machine learning methods once overfitting was controlled. This finding supports prior research indicating that both methods can yield strong abundance‐suitability correlations (Fois et al. 2018; Kulhanek et al. 2011; Osorio‐Olvera et al. 2020; Ready et al. 2010).

We found substantial differences in the abundance‐suitability relationships among some of the modeling methods, particularly the machine learning approaches. These variations can be attributed to the way each method captures and adjusts species' responses to environmental data. For instance, Random Forest (RDF) tends to overfit presence data, resulting in highly sensitive predictions (Fitzgibbon et al. 2022). In contrast, MaxLike (MLK) effectively distinguishes high‐suitability areas when presence data aligns with abundance patterns (Fitzpatrick et al. 2013; Merow and Silander 2014; Royle et al. 2012). Gaussian (GAU) models also performed well, offering smooth, ecologically plausible responses by flexibly modeling predictor interactions (Golding and Purse 2016). This contrasts with the more irregular surfaces generated by BRT (Elith et al. 2006; Wenger and Olden 2012). Although MLK and GAU exhibited high correlation values, other methods, such as Minimum Volume Ellipsoid (MVE) and MaxEnt (MX), which did not demonstrate significant differences in this study, have also been effective in predicting species abundance in previous research (Osorio‐Olvera et al. 2020; Williams et al. 2021). The objective of this study is not to identify a single superior method, but rather to compare different approaches within the study system, which is characterized by diverse habitats, high dispersal capacity, and migratory behavior. While ensemble modeling has shown promise (de la Fuente et al. 2021; Souza et al. 2022), our findings caution against combining outputs from conceptually distinct models—such as MX and MLK—as this may lead to conflicting ecological interpretations (Phillips et al. 2006; Royle et al. 2012).

Another aspect we aimed to investigate was whether the relationship between abundance and environmental suitability could be affected by the representativeness of presence records within the species' range and/or the temporal alignment of environmental variables with the reproductive season. However, we did not observe significant differences in correlation values based on the temporal alignment of environmental variables or the spatial extent of presence data, suggesting that species consistently responded to climatic variables. This led to stable relationships regardless of whether the data came from full ranges or breeding areas or whether annual or seasonal predictors were used. This robustness aligns with prior research on birds and mammals (Weber et al. 2017; Tôrres et al. 2012; Wilson et al. 2018), though studies on plants suggest variability under specific gradients (Baer and Maron 2020). Nevertheless, using time‐averaged abundance data may have obscured finer spatiotemporal patterns because recent evidence shows stronger correlations when temporal variation in abundance is low (Monnier‐Corbel et al. 2023). Future research should consider temporal abundance fluctuations and alternative calibration strategies, such as using maximum abundance thresholds, when such data become available.

Several limitations must be acknowledged. First, all models were correlative and did not incorporate spatial random effects, which could account for residual spatial autocorrelation in abundance data. Future efforts could incorporate spatially explicit frameworks such as those based on stochastic partial differential equations (SPDE) implemented in R‐INLA (Lindgren et al. 2011), which model spatial structure directly and are useful when spatial autocorrelation is present in the residuals. Second, we did not account for imperfect detection in our models. Site‐occupancy and N‐mixture models explicitly consider detection probability and can provide more accurate estimates of occurrence and abundance, particularly when detection is imperfect or variable across space or time (MacKenzie et al. 2002; Royle 2004). These approaches, however, require specific data structures (e.g., detection/non‐detection or repeated counts) that were not available for our modeling framework. Future research could benefit from incorporating detection‐adjusted models when suitable data are available. Third, we did not specifically assess the performance of models in areas of highest observed abundance. In such zones, variation in abundance may be high even within narrow suitability ranges, weakening the suitability‐abundance relationship. These regions, however, often represent critical population cores and are of particular relevance to conservation strategies. Future studies should evaluate how well presence‐only models can capture abundance patterns in these areas. Fourth, although we mitigated spatial sampling bias by filtering, this approach may eliminate ecologically relevant data where species naturally cluster. Incorporating bias covariates could preserve this information; however, it poses challenges such as multicollinearity and limited transferability (Inman et al. 2021; Dubos et al. 2022). Finally, we did not consider species‐specific BAM configurations (Biotic‐Abiotic‐Movement framework; Soberon and Peterson 2005), which could affect how accessibility constraints and biotic interactions influence abundance patterns. For example, some species may avoid high‐suitability areas due to dispersal limitations (Yañez‐Arenas et al. 2020), while others, such as the Bachman's sparrow and lark bunting, are affected by fine‐scale biotic or climatic factors (Fish et al. 2018; Wilson et al. 2018). Furthermore, using long‐term average abundance data may obscure interannual variation that could reveal dynamic responses to changing conditions (Monnier‐Corbel et al. 2023). Future studies should incorporate temporal fluctuations in abundance and, when available, explore spatially explicit models that integrate biotic and land‐use data. This would allow for a better identification of areas of high abundance, which are often key to conservation planning. It is essential to assess how well simple presence‐based ENMs capture these areas to improve their utility in guiding resource allocation and prioritization efforts in biodiversity management.

This study contributes to the ongoing debate on the predictive capability of ecological niche models and species distribution models in estimating species abundance (Dallas et al. 2017; de la Fuente et al. 2021; Lopes et al. 2022; Osorio‐Olvera et al. 2020; Santini et al. 2019). Our results show that all evaluated methods can partially capture the spatial distribution of species abundance, as evidenced by positive and significant abundance‐suitability correlations for most species (74.4%). These findings highlight the potential of ENM to infer abundance patterns. Additionally, we note that similar trends were maintained within taxonomic groups (e.g., Passerellidae), which could support the generalizability of these findings. Finally, the lack of significant differences in abundance‐suitability relationships when using full‐range versus reproductive‐area data, or annual versus seasonal environmental variables, suggests that simpler, commonly used data inputs may suffice in many contexts, such as conservation planning, carrying capacity estimation, invasive species management, and public health surveillance, particularly when data are limited or fine‐scale inputs are unavailable. These advances understanding by demonstrating that reliable abundance predictions can be achieved without overcomplicating input requirements, providing a practical path forward for ecological and conservation applications. Abundance predictions can aid in estimating carrying capacity in specific sites, thereby enhancing our understanding of species distribution and community dynamics (VanDerWal et al. 2009; Williams et al. 2021). Moreover, abundance proxies derived from modeled environmental suitability are valuable when data are limited, informing conservation strategies and facilitating invasive species management (Malone et al. 2018; Wilson et al. 2011). Understanding species responses to local conditions is crucial for conservation biology, enabling predictions of invasive species impacts (Kulhanek et al. 2011). Additionally, abundance‐suitability relationships can inform public health efforts, such as in Chagas disease vector control in Latin America, where the spatial distribution of abundance aligns with the niche centrality hypothesis (Altamiranda‐Saavedra et al. 2020).

## 5 Conclusion

Our results suggest that the spatial distribution of abundance could be partially explained by local environmental conditions, confirming the basis of ecological niche theory (Maguire 1973). These results offer future applications in predicting abundance within ecology and conservation, such as estimating the carrying capacity in geographic areas, including this information in predictive approaches of community composition, or establishing conservation strategies.

## Author Contributions

**Jazmín Escobar‐Luján:** conceptualization (equal), data curation (equal), formal analysis (equal), methodology (equal), resources (equal), writing – original draft (equal), writing – review and editing (equal). **Fabricio Villalobos:** conceptualization (equal), methodology (equal), supervision (equal), writing – review and editing (equal). **Adolfo G. Navarro‐Sigüenza:** conceptualization (equal), methodology (equal), supervision (equal), writing – review and editing (equal). **Carlos Yañez‐Arenas:** conceptualization (equal), methodology (equal), supervision (equal), writing – review and editing (equal).

## Conflicts of Interest

The authors declare no conflicts of interest.

## Supporting information


**Appendix S1:** List of species distribution modeling methods used, along with their main applied parameters in this study.
**Table S1:** List of bird species from North America included in the study.
**Figure S1:** Rho values of the relationships between suitability modeled by methods and abundance for species of the Passerellidae family.

## Data Availability

The data that support the findings of this study are openly available in Figshare at: https://figshare.com/articles/dataset/_b_Comparative_analysis_ENM_methods_for_abundance‐suitability_relationship_b_/28200344/1?file=51654878.
