# Supplementary material for: Comparative Analysis of Correlative Modeling Methods in Predicting North American Bird Abundance
Source: Ecol Evol. 2026 Aug 3;16(8):e71982. doi: 10.1002/ece3.71982 (PMC13431883; doi:10.1002/ece3.71982)
Supplement: Supplementary file 1 — Appendix S1: List of species distribution modeling methods used, along with their main applied parameters in this study. Table S1: List of bird species from North America included in the study. Figure S1: Rho values of the relationships between suitability modeled by methods and abundance for species of the Passerellidae family. [file ECE3-16-e71982-s001.docx]

**Supporting information**

**S1 Table.** List of bird species from North America included in the study. M: migratory species, NM: non-migratory species. N: number of presence records used. For migratory species, the number of presence records within the breeding area is provided (N rep).

| **Family** | **Specie** | **Migratory** | **N (N rep)** |
| --- | --- | --- | --- |
| Anatidae | *Aix sponsa* | M | 1061(162) |
| Apodidae | *Chaetura pelagica* | M | 1073(992) |
| Calcariidae | *Rhynchophanes mccownii* | M | 457(184) |
| Caprimulgidae | *Antrostomus carolinensis* | M | 856(589) |
|  | *Antrostomus vociferus* | M | 1042(872) |
|  | *Phalaenoptilus nuttallii* | M | 905(601) |
| Cardinalidae | *Passerina amoena* | M | 812(719) |
|  | *Passerina cyanea* | M | 1144(765) |
|  | *Pheucticus ludovicianus* | M | 1246(562) |
|  | *Pheucticus melanocephalus* | M | 655(556) |
|  | *Piranga olivacea* | M | 1064(710) |
|  | *Piranga rubra* | M | 837(426) |
|  | *Spiza americana* | M | 1031(822) |
| Certhiidae | *Certhia americana* | M | 1332(946) |
| Charadriidae | *Charadrius montanus* | M | 305(138) |
| Corvidae | *Corvus ossifragus* | NM | 468 |
|  | *Cyanocitta cristata* | NM | 1133 |
|  | *Gymnorhinus cyanocephalus* | NM | 484 |
|  | *Nucifraga columbiana* | NM | 569 |
|  | *Pica nuttalli* | NM | 66 |
| Cuculidae | *Coccyzus americanus* | M | 1318(1088) |
|  | *Coccyzus erythropthalmus* | M | 884(811) |
| Fringillidae | *Haemorhous cassinii* | M | 617(591) |
|  | *Coccothraustes vespertinus* | M | 794(650) |
|  | *Spinus lawrencei* | M | 221(165) |
| Hirundinidae | *Progne subis* | M | 941(826) |
|  | *Stelgidopteryx serripennis* | M | 859(777) |
| Icteridae | *Agelaius tricolor* | NM | 194 |
|  | *Dolichonyx oryzivorus* | M | 1307(1139) |
|  | *Euphagus cyanocephalus* | M | 814(556) |
|  | *Icteria virens* | M | 1059(829) |
|  | *Icterus bullockii* | M | 750(601) |
|  | *Icterus galbula* | M | 1100(645) |
|  | *Quiscalus major* | NM | 192 |
|  | *Quiscalus quiscula* | M | 1203(1203) |
|  | *Sturnella neglecta* | M | 932(916) |
|  | *Xanthocephalus xanthocephalus* | M | 1155(781) |
| Mimidae | *Dumetella carolinensis* | M | 1121(718) |
|  | *Oreoscoptes montanus* | M | 844(702) |
|  | *Toxostoma rufum* | M | 1070(1032) |
|  | *Toxostoma redivivum* | NM | 118 |
| Odontophoridae | *Oreortyx pictus* | NM | 238 |
| Paradoxornithidae | *Chamaea fasciata* | NM | 157 |
| Paridae | *Baeolophus bicolor* | NM | 1038 |
|  | *Baeolophus inornatus* | NM | 127 |
|  | *Baeolophus ridgwayi* | NM | 319 |
|  | *Poecile carolinensis* | NM | 781 |
|  | *Poecile gambeli* | NM | 508 |
| Parulidae | *Geothlypis formosa* | M | 838(618) |
|  | *Helmitheros vermivorum* | M | 748(482) |
|  | *Limnothlypis swainsonii* | M | 546(446) |
|  | *Oreothlypis virginiae* | M | 48(31) |
|  | *Parkesia motacilla* | M | 1092(771) |
|  | *Protonotaria citrea* | M | 817(585) |
|  | *Setophaga americana* | M | 770(533) |
|  | *Setophaga cerulea* | M | 658(517) |
|  | *Setophaga citrina* | M | 710(472) |
|  | *Setophaga discolor* | M | 620(410) |
|  | *Setophaga dominica* | M | 583(221) |
|  | *Setophaga nigrescens* | M | 561(392) |
|  | *Setophaga occidentalis* | M | 415(252) |
|  | *Setophaga pinus* | M | 859(406) |
|  | *Vermivora cyanoptera* | M | 823(604) |
| Passerellidae | *Ammodramus henslowii* | M | 295(230) |
|  | *Ammodramus maritimus* | M | 137(134) |
|  | *Ammodramus savannarum* | M | 1378(1087) |
|  | *Calamospiza melanocorys* | M | 859(603) |
|  | *Chondestes grammacus* | M | 806(523) |
|  | *Peucaea aestivalis* | M | 221(218) |
|  | *Pipilo chlorurus* | M | 672(517) |
|  | *Pipilo erythrophthalmus* | M | 994(880) |
|  | *Spizella breweri* | M | 838(512) |
|  | *Spizella pusilla* | M | 1017(979) |
| Phasianidae | *Centrocercus urophasianus* | NM | 498 |
|  | *Dendragapus fuliginosus* | NM | 326 |
|  | *Tympanuchus cupido* | NM | 261 |
| Picidae | *Dryocopus pileatus* | NM | 1492 |
|  | *Melanerpes carolinus* | NM | 998 |
|  | *Melanerpes erythrocephalus* | M | 1054(113) |
|  | *Melanerpes lewis* | M | 580(112) |
|  | *Picoides albolarvatus* | NM | 141 |
|  | *Picoides borealis* | NM | 209 |
|  | *Sphyrapicus nuchalis* | M | 743(454) |
|  | *Sphyrapicus thyroideus* | M | 511(189) |
| Podicipedidae | *Aechmophorus clarkii* | M | 300(115) |
|  | *Aechmophorus occidentalis* | M | 381(213) |
| Rallidae | *Rallus elegans* | M | 311(92) |
|  | *Rallus limicola* | M | 914(436) |
| Scolopacidae | *Numenius americanus* | M | 299(47) |
|  | *Scolopax minor* | M | 1172(840) |
| Sittidae | *Sitta pusilla* | NM | 398 |
| Strigidae | *Megascops asio* | NM | 926 |
| Threskiornithidae | *Plegadis chihi* | M | 719(56) |
| Trochilidae | *Archilochus alexandri* | M | 508(333) |
|  | *Archilochus colubris* | M | 1256(943) |
|  | *Calypte anna* | M | 328(37) |
|  | *Selasphorus sasin* | M | 59(22) |
| Troglodytidae | *Cistothorus palustris* | M | 777(390) |
|  | *Cistothorus platensis* | M | 983(412) |
|  | *Thryomanes bewickii* | M | 576(576) |
|  | *Troglodytes aedon* | M | 1848(1496) |
| Turdidae | *Hylocichla mustelina* | M | 819(603) |
| Tyrannidae | *Contopus virens* | M | 1124(768) |
|  | *Empidonax difficilis* | M | 309(236) |
|  | *Empidonax oberholseri* | M | 873(607) |
|  | *Empidonax occidentalis* | M | 483(489) |
|  | *Empidonax traillii* | M | 1231(1000) |
|  | *Empidonax virescens* | M | 1057(843) |
|  | *Empidonax wrightii* | M | 755(463) |
|  | *Myiarchus crinitus* | M | 789(564) |
|  | *Tyrannus forficatus* | M | 664(450) |
|  | *Tyrannus verticalis* | M | 1031(819) |
| Vireonidae | *Vireo bellii* | M | 433(332) |
|  | *Vireo flavifrons* | M | 932(602) |
|  | *Vireo gilvus* | M | 1078(863) |
|  | *Vireo griseus* | M | 667(526) |
|  | *Vireo olivaceus* | M | 1609(1013) |
|  | *Vireo vicinior* | M | 399(348) |

**
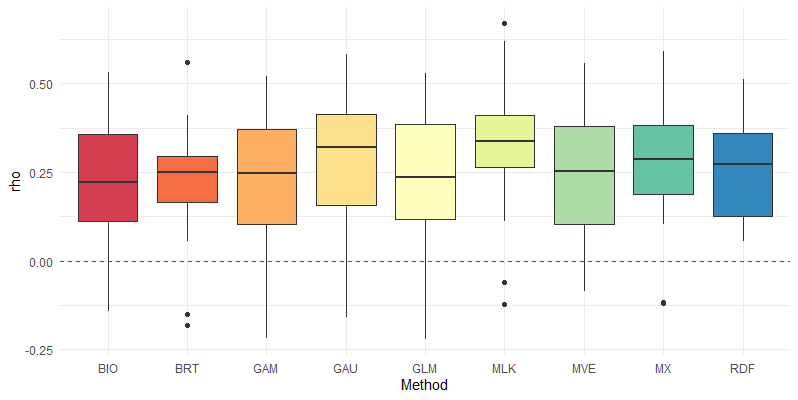
**

**S1 Figure. Rho values of the relationships between suitability modeled by methods and abundance for species of the Passerellidae family.** Abbreviations: BIO: Bioclim, BRT: Boosted regression tree, GAM: Generalized additive models, GAU: Gaussian models, GLM: Generalized linear models, MLK: Maxlike, MVE: Minimum volume ellipsoids, MX: Maxent, RDF: Random Forest.

**Appendix S1. List of species distribution modeling methods used, along with their main applied parameters in this study. Modeling approach, acronym, parameters used, and R package employed.** This appendix provides a detailed description of the SDM/ENM methods applied in this study, along with the primary parameters and corresponding R packages used for model calibration and evaluation.

| **Modeling approach** | **Methods Name** | **Acronym** | **Parameters used** | **R package** | **References** |
| --- | --- | --- | --- | --- | --- |
| Statistical or regression | Generalized linear models | GLM | Method: Linear regression model for binomial data, formula (linear responses) | stats | R Core Team 2022 |
|  | Generalized additive models | GAM | Method: Non-linear model using splines for flexible fits, formula (linear and quadratic responses) smooth terms | gam | Hastie (2018) |
|  | Gaussian models | GAU | Method (Laplace Approximation), Laplace Approximation for modeling probability densities of species presence | GRaF | Golding (2014) |
| Similarity or envelope | BIOCLIM | BIO | Default (presence records) | dismo | Hijmans et al. (2017) |
|  | Minimum volume ellipsoids | MVE | Covariance matrix with 99% of presence records | ntbox | Osorio‐Olvera et al. (2020) |
| Machine learning | Maximum entropy modelling, MAXENT | MX | Features (L, Q and P), reg_mult (1 to 4), iterations (1000) | maxnet | Phillips (2017) |
|  | MaxLike | MLK | Formula (linear and quadratic responses) | maxlike | Royle et al. (2012) |
|  | Random Forest | RDF | Trees (1000) | randomForest | Liaw & Wiener (2002) |
|  | Boosted Regression Tree | BRT | Trees (1000) | dismo | Hijmans et al. (2017) |

Presence records were divided into four spatially independent blocks using the ENMEval package (Muscarella et al., 2014), ensuring spatial independence between calibration and evaluation datasets. In each iteration, two diagonally opposite blocks were used for model calibration, and the other two for evaluation. Specifically, in the first iteration, blocks 1 and 3 were employed for calibration, while blocks 2 and 4 were used for evaluation. In the second iteration, the calibration and evaluation blocks were swapped, with blocks 2 and 4 used for calibration and blocks 1 and 3 for evaluation. For each species, 10,000 background points were randomly generated within the defined M area to represent the available environmental background. These background points were then assigned to the same spatial blocks as the presence records, with blocks 1 and 3 used for model generation in the first iteration and blocks 2 and 4 in the second. Each model generated during the iterations was evaluated using partial ROC analysis and omission rates, with an acceptable error margin of 5% in both metrics (Peterson et al., 2008). After training and evaluating the models in each iteration, final predictions were generated by training a model using all combined presence records from all blocks. These final models were built using the same configurations and methods for each species, and predictions were made using the same environmental variables across the entire study area, producing raster maps of species distribution predictions.

**References**

Golding, N. (2014). GRaF: Species distribution modelling using latent Gaussian random fields. R package version 0.1-12.

Hastie, T. J. (2018). Generalized additive models. In Statistical models in S (pp. 249-307). Routledge.

Hijmans, R. J., Phillips, S., Leathwick, J., Elith, J., & Hijmans, M. R. J. (2017). Package ‘dismo’. Circles, 9(1), 1-68.

Liaw, A., & Wiener, M. (2002). Classification and regression by randomForest. R news, 2(3), 18-22.

Muscarella, Robert., Galante, P. J., Soley-Guardia, Mariano., Boria, R. A., Kass, J. M., Uriarte, M., & Anderson, R. P. (2014). ENMeval: An R package for conducting spatially independent evaluations and estimating optimal model complexity for Maxent ecological niche models. Methods in Ecology and Evolution, 5, 1198–1205. https://doi.org/10.1111/2041-210x.12261

Osorio‐Olvera, L., Lira‐Noriega, A., Soberón, J., Peterson, A. T., Falconi, M., Contreras‐Díaz, R. G., ... & Barve, N. (2020). ntbox: An r package with graphical user interface for modelling and evaluating multidimensional ecological niches. Methods in Ecology and Evolution, 11(10), 1199-1206.

Peterson, A. T., Papeş, M., & Soberón, J. (2008). Rethinking receiver operating characteristic analysis applications in ecological niche modeling. Ecological Modelling, 213(1), 63–72.

Phillips, S. (2017). Maxnet: Fitting ‘maxent’species distribution models with ‘glmnet’. R package version 0.1, 2.

Royle, J. A., Chandler, R. B., Yackulic, C., & Nichols, J. D. (2012). Likelihood analysis of species occurrence probability from presence‐only data for modelling species distributions. Methods in Ecology and Evolution, 3(3), 545-554.
